# *Streptococcus mutans* promotes tumor progression in oral squamous cell carcinoma

**DOI:** 10.7150/jca.73310

**Published:** 2022-09-21

**Authors:** Ming-Shao Tsai, Yu-Yen Chen, Wen-Cheng Chen, Miao-Fen Chen

**Affiliations:** 1 Department of Otolaryngology & Head and Neck Surgery, Chang Gung Memorial Hospital, Chiayi, Taiwan.; 2 Chang Gung University, College of Medicine, Taiwan.; 3 Department of Radiation Oncology, Chang Gung Memorial Hospital, Chiayi, Taiwan.; 4 Department of Radiation Oncology, Chang Gung Memorial Hospital, Linko, Taiwan.; Ming-Shao Tsai and Yu-Yen Chen contributed equally to this work.

**Keywords:** *S.mutans*, aggressiveness, IL-6, oral SCC

## Abstract

Oral squamous cell carcinoma (OSCC) is an aggressive head and neck cancer. Evidence showed that some pathogenic bacteria are associated with periodontitis and oral cancer. The change in oral microbiome composition and the role of the specific periodontal pathogen *Streptococcus mutans* in OSCC were investigated. We analyzed the microbiome of oral biofilms to identify if the oral microbiome composition was associated with OSCC. The role of *S. mutans* with clinical prognosis for OSCC was also examined. We further examined the role of *S. mutans* infection in OSCC progression in preclinical experiments. The microbiome assay by oral biofilms revealed that there was different microbiota composition between OSCC patients and health participants. Furthermore, the microbiota profiles showed that* S. mutans* abundance was associated with the development of OSCC development. Using the 16S rRNA PCR analysis, the presence of *S. mutans* was associated with advanced clinical stage and poor disease control. Furthermore, in the 4-nitroquinoline 1-oxide-induced mouse model, the presence of *S. mutans* was associated with elevated invasive oral cancer incidence. By cellular and xenograft tumor model using oral cancer cells, *S. mutans* infection was associated with the increased tumor aggressiveness, the epithelial-mesenchymal transition and interleukin-6 (IL-6) production; it also correlated with the recruitment of myeloid-derived-suppressor cells. When IL-6 signaling inhibited, the effects of *S. mutans* on tumor aggressiveness were attenuated. In conclusion, *S. mutans* may have the additive effect on oral cancer development and progression. Good oral hygiene to eradicate *S. mutans* or targeting IL-6 signaling could be a promising strategy for OSCC associated with *S.mutans* infection.

## Introduction

Oral squamous cell carcinomas (OSCC) are a major cause of head and neck cancer morbidity and mortality 1. There is half of patients with high-risk disease experiencing loco-regional recurrence and accounting for the majority of deaths. Smoking betel nut chewing, and poor oral hygiene are major risk factors for OSCC 2, 3. Nowadays, potential biomarkers are under investigation in order to develop biomarker-guided personalized treatment.

Increasing evidence showed that microorganisms are linked to a significant number of human cancers 4, 5. Pathogenic bacterial colonization is highly correlated with inflammation and cancer progression. Poor oral health drives dysbiosis of the microbiome and is associated with dysplasia and carcinogenesis in the head and neck cancer (HNC) 6, 7. Dysbiosis can facilitate cancer through cell proliferation and oncogene activation, induction of chronic inflammation, and lead to impaired local/ systemic immune responses with breakdown of mucosal barriers. Chronic inflammation is reported to promote the development of various tumors, including HNC. Microbes are reported to trigger proinflammatory immune circuits and elicit immunosuppressive responses 8. The oral cavity harbors the complex and diverse microbiomes, which is relevant to the local microenvironment and tumor growth and spread for HNC 6, 7. Recent studies have pointed to the link between inflammation periodontal disease, and the potential contribution of microorganisms to the development of OSCC 9, 10. Pathogenic bacteria in oral biofilms contribute to the development of dental caries, periodontitis, and oral cancer. Lactobacillus spp. and Streptococcus spp. are the main causative organisms leading to dental caries. Streptococcus mutans is one of the bacteria associated with tooth decay, and the main cariogenic microbes among Streptococcus spp. 11, 12. Although *S. mutans* is reported to have a unique virulence property and be a cariogenic organism, but the centrality of *S. mutans* to OSCC is still debated. We propose that the organism is perhaps an important predisposing factor in the microenvironment that directs the development or progression of OSCC. A better understanding of the role of the oral microbiome may allow for the development of novel strategies to treat and predict prognosis for OSCC. Therefore, we examined the association of oral microbiome composition with OSCC and elucidated the effects of *S. mutans* on the tumor characteristics.

## Materials and methods

### Study cohort

The study cohort consisted of 82 patients with OSCC who received curative treatment at our department in accordance with the recommendations of the oncology team of our hospital from 2018 to 2020. The paraffin-embedded tissues obtained at the time of diagnosis from 82 patients were collected for immunochemical analysis and DNA samples for qPCR analysis. The clinical parameters of OSCC patients were recorded in Table 1. The presence of S.mutans was calculated as differences in Ct (cycle threshold value) between PCR using universal primers and *S.mutans* specific primer, e.g., Δ Ct = (Ct for S.mutans - Ct for universal 16S rDNA).To assess the predictive value of the S. mutans, the amount of *S. mutans* was redefined as a binary variable by the mean value 9.35. Accordingly, all OSCC patients were divided into two groups: low (Δ Ct ≥ 9.35) and high (Δ Ct < 9.35) groups. Moreover, oral biofilms were obtained from 52 patients with OSCC and 18 healthy donors. The oral biofilm samples were collected from 36 patients with gross tumor and 18 patients without gross tumor. The oral biofilm samples were collected from each participant before radiotherapy, and obtained by swabbing the dental plaques at the gingival margin on the molars with sterilized toothpicks. The study was approved by the Institutional Review Board of our hospital.

### Immunohistochemical (IHC) Staining and Immunofluorescence (IF) of Tissue Specimens

Formalin-fixed, paraffin-embedded tissues were cut into 5 μm sections, mounted on slides, de-paraffinized with xylene, and dehydrated using a graded ethanol series for IHC. Sections were incubated overnight at 4 °C with antibodies against target proteins. After three PBS washes, the sections were incubated for 10 min with the biotinylated secondary antibody, stained with peroxidase-avidin, and washed in PBS; 3-amino-9-ethylcarbazole solution was then added. The sections were counterstained with hematoxylin. The IHC data for the specimens were assessed using the semi-quantitative immunoreactive score (IRS). The IRS was calculated by multiplying the staining intensity (graded as: 0 = no, 1 = weak, 2 = moderate and 3 = strong staining) and the percentage of positively stained cells (0 = less than 10 % of stained cells, 1 = 11-50% of stained cells, 2 = 51-80% of stained cells and 3 = more than 81 % of stained cells). The criterion for positive staining is a specimen with an IRS scoring grade greater or equal to 2. Frozen tissue specimens were sliced into 5-8 μm sections in a cryostat. The sections were incubated overnight at 4 °C with anti-bodies against target proteins, washed three times with PBS, and incubated for 1 h with fluorescein or Texas Red-conjugated secondary antibodies. The slides were counterstained with DAPI to visualize the nuclei. After two washes with PBST, the specific target proteins were visualized using a fluorescence microscope.

### Determination of16S rDNA in OSCC tissue and quantitative polymerase chain reaction (qPCR) for Streptococcus mutans

We extracted DNA from the mentioned 82 OSCC paraffin-embedded tissues obtained at the time of diagnosis. For amplification, DNA concentrations were adjusted to 8 ng/ml. 16SrDNA samples were amplified using *S. mutans* specific and universal 16S rDNA primers. Furthermore, to examine if the differential expression of S*. mutans between* malignant and non-malignant tissue cancer tissue*,* DNA was extracted from fresh oral cancer specimens of 12 OSCC patients and 12 from non-malignant oral tissues. We determined the amount of S*. mutans* DNA by qPCR assay.

### Microbiome analysis

We extracted DNA from oral biofilm samples. Barcoded amplicons were generated covering the 16S rRNA gene V3-V4 region using 341F/805R primers. The detail was described previously 13. Sequencing of multiplexed pooled libraries was performed on a MiSeq system (Illumina, San Diego, CA, USA). The quality-filtered reads from 70 samples were clustered into operational taxonomic units (OUT). The α-diversity (within-subject diversity) was assessed by richness, and the β-diversity (between-subject diversity) was assessed at the OTU level using unweighted and weighted UniFrac distances. The differential abundances between healthy individuals and patients with OSCC were determined using linear discriminant analysis (LDA) effect size and DESeq2.

### Cell and bacterial cultures

The human oral cancer cell line SCC4 and SCC25 and *S. mutans* were obtained from the Bioresource Collection and Research Center. *S. mutans* was grown in Aerobic BD Difco™ Bacto™ Brain Heart Infusion. OSCC cells were infected with *S. mutan* at a multiplicity of infection of 1:100 for 24 h or 48 h at 37 °C. Infected cells were used for subsequent *in vitro* experiments. Uninfected cells were used as controls. To determine the *in vitro* effects of the anti-IL-6 antibody, cells incubated in the presence of 5 μg/ml IL-6 neutralizing antibodies or isotype antibody for 48 h.

### Animals and experimental design

All experimental procedures involving animals were approved by the Experimental Animal Ethics Committee of our hospital. Six-week-old male C57BL/6 mice were used for the 4-nitroquinoline 1-oxide (4-NQO)-induced tongue tumor model 14, 15. Briefly, A stock solution of the carcinogenic 4-NQO (Sigma, St. Louis, MO, USA) was prepared in propylene glycol to a final concentration of 5 mg/ml. In 4-NQO-treated group, the tongue was stroked and painted with the 4-NQO stock solution three times a week and allowed access to drinking with 4-NQO-containing water for 16 weeks (tumor-induced group), as described previously 13, 15. Mice in the tumor-induced group were randomly divided into two groups: 4NQO and 4NQO+SM. Animals in the 4NQO+SM group were infected with *S. mutans* (200 µL of bacteria at 10^10^ cells/mL) three times per week for 2 weeks prior to 4NQO administration; they then underwent 8 weeks of 4NQO treatment, followed by 10 weeks of bacterial infection (twice per week). Additionally, athymic mice were used to establish oral cancer xenograft models. Human oral cancer cells (1×10^6^ cells per mouse) were subcutaneously injected into the right thigh.

### Small-animal imaging

*In vivo* optical imaging was performed in 4NQO-treated mice using fluorescence molecular tomography to measure oral tumor induction at indicated time points. The fluorescent probe 2-DeoxyGlucosone 750 was used for *in vivo* tumor imaging, based on enhanced glucose uptake in tumor cells, compared to surrounding non-malignant tissues. After imaging, the presence of mouse oral lesions was further evaluated by gross examination of tissue samples.

### Flow cytometry analysis

The single-cell suspensions were blocked and subsequently stained with antibodies against CD44 and ALDH1 to analyze their expression levels using a FACS caliber flow cytometer (BD Biosciences). In addition, we used antibodies specific for CD11b and Gr1 to define MDSC in murine tumors, respectively. FACS analysis was carried out on single-cell suspensions prepared from whole tumors after digestion and immunostaining for CD11b/ Gr1 with fluorescence-labeled monoclonal antibodies (BD Pharmingen).

## Results

### Oral microbiome composition in OSCC

**Figure 1:**
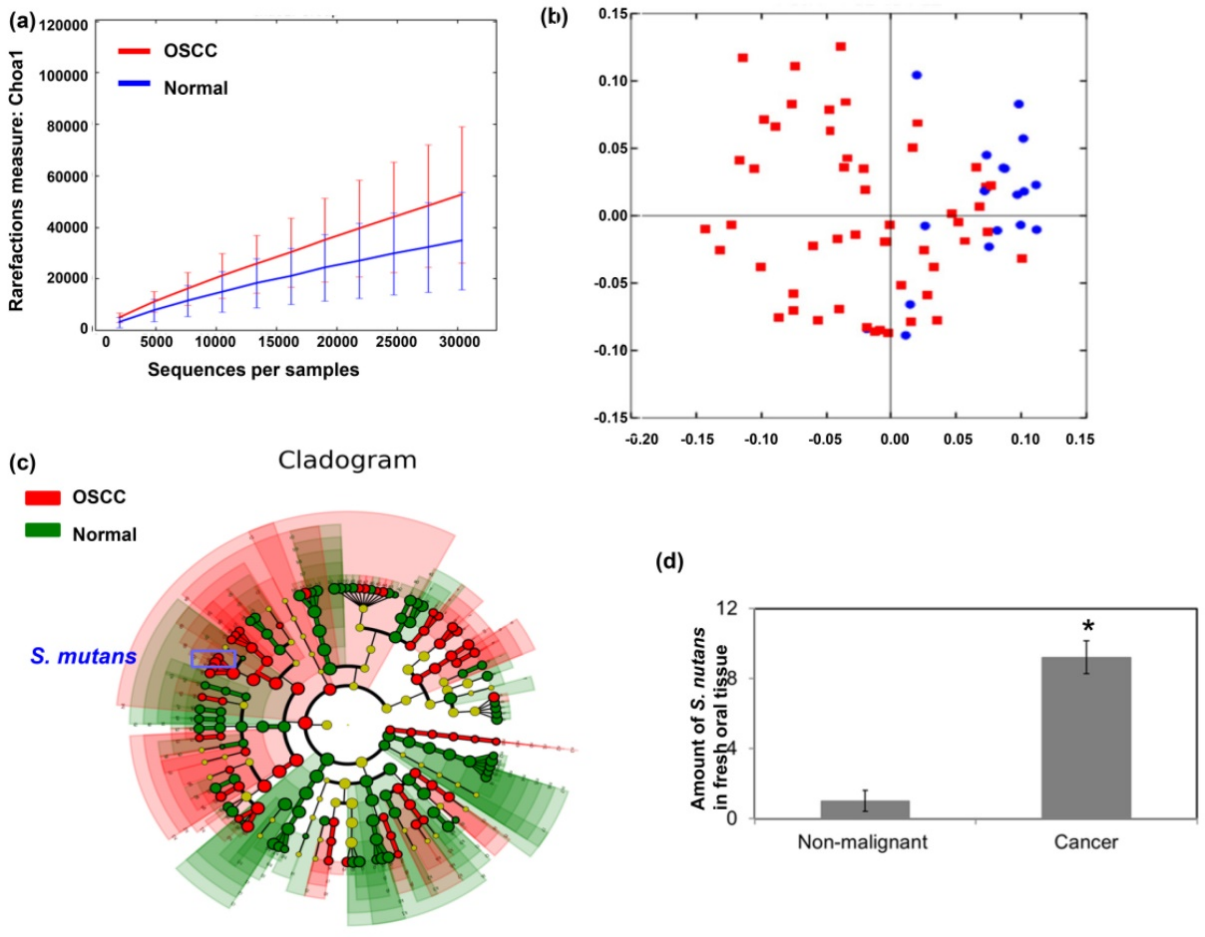
***Streptococcus mutans* is enriched in oral squamous cell carcinoma.** Microbiome assay for oral specimens in OSCC patients are shown with **(a)** α-diversity and **(b)** β-diversity in color. Red, OSCC; Blue, healthy. **(c)** Cladogram showing differentially enriched species in the oral microbiome of patients with OSCC and matched healthy individuals. Species significantly associated with OSCC are shown in color. Red, OSCC > healthy; Green, OSCC < healthy. **(d)** *S. mutans* 16S rDNA levels in fresh oral tissue specimen of patients with OSCC and matched healthy individuals, as determined by quantitative polymerase chain reaction.

To investigate the correlation of the oral microbiome composition with OSCC, oral biofilms were obtained from patients with OSCC and healthy volunteers. Figure 1a demonstrated that patients with OSCC had higher species richness than healthy individuals. Furthermore, there was a significant difference in overall oral microbial composition between patients with OSCC and healthy individuals (*p*=0.001) (Fig. 1b). The linear discriminant analysis (LDA) was used to identify differentially enriched species. Some *Streptococcus* species, such as *Streptococcus mutans,* were more abundant in oral biofilms from patients with OSCC than that from healthy volunteers (Fig. 1c). Moreover, for *S. mutans*, there was significant difference between cancer and normal group by DESeq2 (Cancer versus Normal: Fold change 23.895; *p* <0.0001). To confirm that *S. mutans* was more frequent in cancer tissues, *S. mutans* 16S rDNA levels were measured in fresh oral tissue specimens by qPCR. Figure 1d showed that *S. mutans* 16S rDNA was present to be more abundant in cancer specimens than in non-malignant oral samples.

### S. mutans infection associates with poor prognosis in OSCC

**Figure 2:**
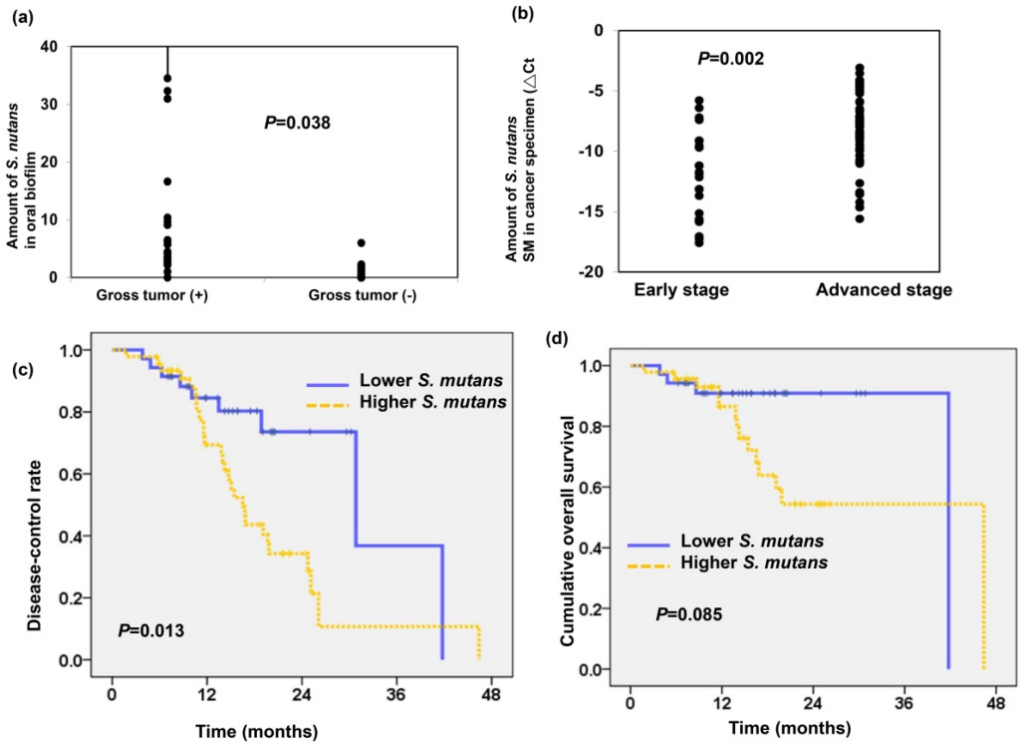
***S. mutans* infection associates with poor prognosis in OSCC. (a)** The levels of *S.mutans* detected in oral biofilm correlated with the tumor burden. **(b)** The presence of *S.mutans* by qPCR using OSCC paraffin block correlated with clinical stage. Cumulative disease-control rate **(c),** and overall survival **(d)** of patients with OSCC according to the presence of *S.mutans* by qPCR*.*

As shown in Figure 2a, we found the amount of *S.mutans* detected in oral biofilm was higher in patients with gross tumor compared to those without gross tumor (*P*=0.038). To further assess the relationship between *S.mutans* in cancer tissue and OSCC prognosis, we examined the presence of *S.mutans* by qPCR using OSCC specimens. The DNA extracted from 82 OSCC paraffin blocks obtained at diagnosis were used for the detection of *S.mutans* infetion. As shown in Figure 2b, there was a higher amount of S.mutans detected in cancer specimen for locally advanced OSCC compared to early stage (△Ct -12.06± 0.72 in the early stage group and △Ct -8.2± 0.43 in the advanced stage group; P=0.002). The clinical characteristics of patients with OSCC are summarized in Table 1. In total, 46 of 82 specimens from patients with OSCC (56%) were associated with a higher amount of *S.mutan*s. Importantly, the presence of *S.mutans* was significantly associated with poor differentiation, advanced disease and higher risk of developing disease failure. We further analyzed the role of *S.mutans* infection in predicting cumulative disease failure and survival. Figure 2c-d revealed that a higher amount of *S.mutans* in oral cancer specimens correlated with low disease-control rate (p=0.013) but not significantly related to cumulative overall survival (p=0.085). These findings suggest that *S.mutans* infection contributes to tumor aggressiveness and associated with poor disease control in patients with OSCC.

### S. mutans infection promotes oral carcinogenesis in 4NQO-induced mouse tumors

**Figure 3:**
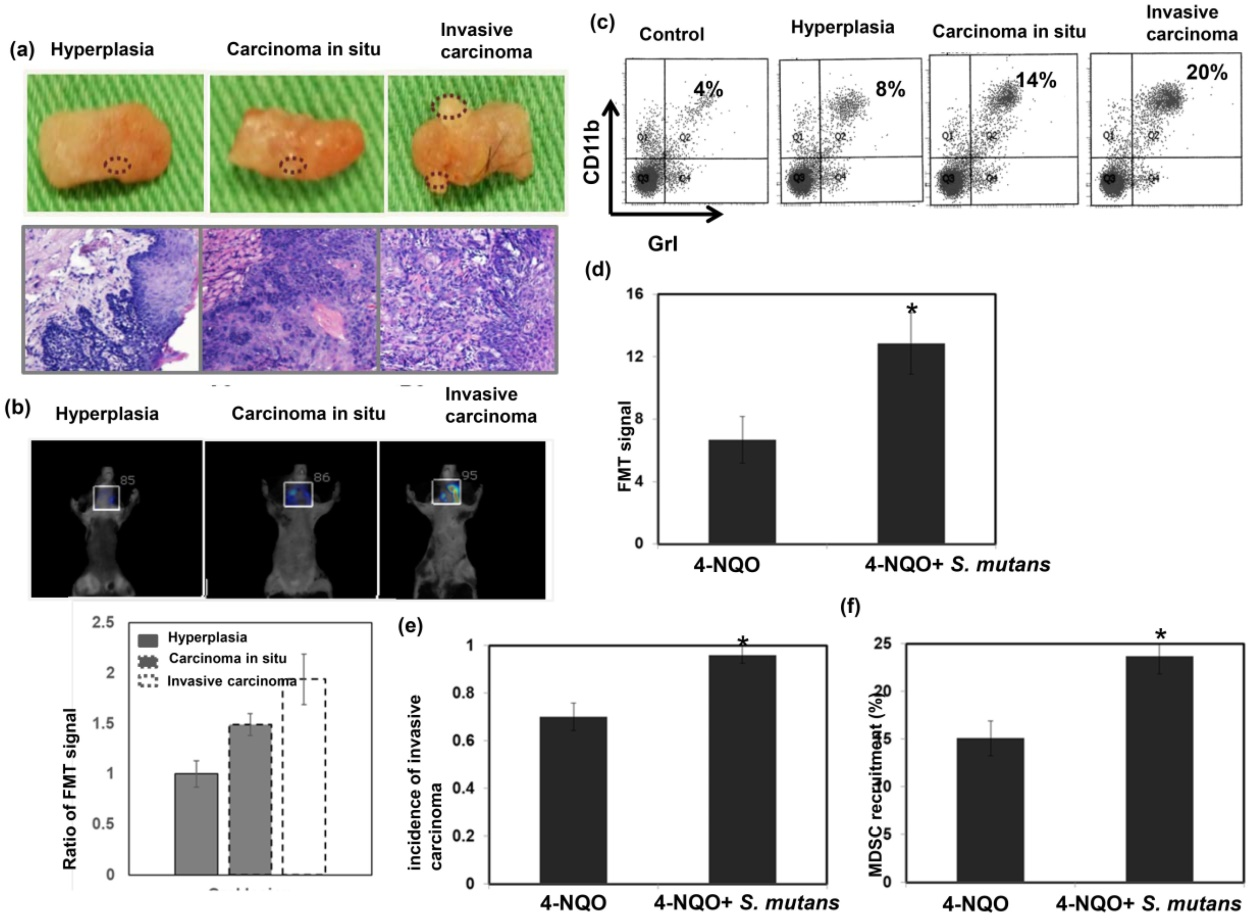
***S. mutans* infection promotes tumor progression in 4NQO-induced mouse tumors.** We evaluated oral tumor formation in 4-NQO-induced tumor model. **(a)** Gross examination of lesions from the animals treated with 4NQO or vehicle for 16 weeks. 4NQO treatment promoted the development of hyperplasia, carcinoma *in situ*, and invasive carcinoma. **(b)** Representative fluorescence molecular tomography images from 4NQO-treated mice that developed hyperplasia, carcinoma *in situ*, or invasive carcinoma. *, *p*<0.05. **(c)** CD11b^+^Gr1^+^ myeloid-derived suppressor cell (MDSC) infiltration was determined by flow cytometry. *, *p*<0.05. Moreover, effects of *S. mutans* infection on oral tumor formation in 4-NQO-treated mice were determined by fluorescence molecular tomography analysis of glucose uptake **(d)**, increased incidence of developing invasive carcinoma **(e)** and flow cytometry analysis of MDSCs **(f)**. Data are presented as means ± standard errors of the mean.

To further elucidate the relationship between *S. mutans* infection and oral tumorigenesis, a 4NQO-induced oral-tongue cancer mouse model was established. Lesions of oral tongue were observed in 4NQO-treated mice after 4NQO-induced tumor period; most lesions constituted hyperplasia/papilloma, severe dysplasia/carcinoma *in situ*, and invasive carcinoma (Fig. 3a). The presence of lesions at 12 weeks after the completion of 4NQO treatment was examined by fluorescence molecular tomography (Fig. 3b). Oral lesions with invasive carcinoma exhibited significantly enhanced glucose uptake, compared to benign tumors (hyperplasia or papilloma). This study also examined the link between tumor progression and the presence of MDSCs in the lesions; significantly higher numbers of MDSCs were present in tumors from mice with invasive carcinomas, compared to tumors from mice with benign lesions (Fig. 3c). Importantly, as shown in Figure 3d-f, *S. mutans* infection was associated with the increased incidence of developing invasive carcinoma, enhanced glucose uptake on lesions, and MDSC recruitment in 4NQO-treated mice.

### S. mutans enhances oral cancer cell invasion *in vitro* and *in vivo*

**Figure 4:**
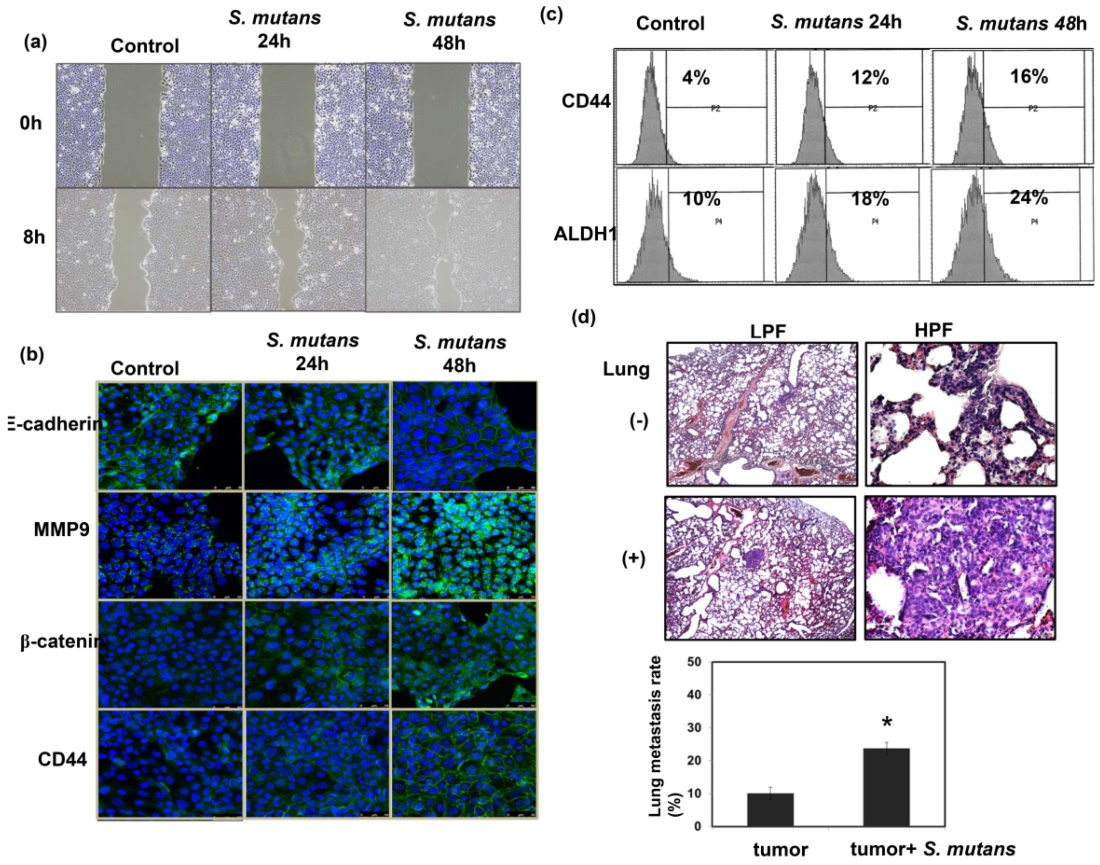
***S. mutans* enhances oral cancer cell invasion and stemness. (a)** Effects of *S. mutans* infection on oral cancer cell invasion. **(b)** Effects of *S. mutans* infection on EMT-associated protein levels. Changes in E-cadherin, matrix metalloproteinase-9, CD44, and β-catenin protein levels were determined by immunofluorescence (DAPI, blue; target proteins, green) in SCC4 cells. **(c)** Effects of *S. mutans* infection on expression of CSC-related proteins CD44 and ALDH1 in SCC25 cells, as determined by flow cytometry. **(d)** Effects of *S. mutans* infection on the rate of lung metastasis in tumor-bearing mice were evaluated by histologic examination.

To examine the role of *S. mutans* infections in cancer cell invasiveness, *in vitro* wound healing assays were performed using *S. mutans*-infected human oral SCC cells. *S-mutans*-infected SCC4 cells exhibited a profound enhancement of invasive potential, compared with control cells (Fig. 4a). Epithelial-mesenchymal transition (EMT) plays a critical role in cancer cell invasion and metastasis. Notably, *S. mutans*-infected OSCC cells exhibited enhanced EMT-associated characteristics, including elevated β-catenin and matrix metalloproteinase-9 levels, accompanied by reduced E-cadherin expression (Fig. 4b). Cancer stem cells (CSCs) are critical for tumor progression in various cancers. *S. mutans*-infected human OSCC cells exhibited elevated expression levels of the CSC markers CD44 and ALDH1 (Fig. 4b-c). We further determined the metastatic ability by examining lung metastasis after intravenous injection of 1×10^7^ cells of each tumor type in the mice. As shown in Figure 4d, *S. mutans* infection was associated with the increased incidence of lung metastasis.

### Role of S. mutans infection in the expressions of IL-6 for oral cancer

**Figure 5:**
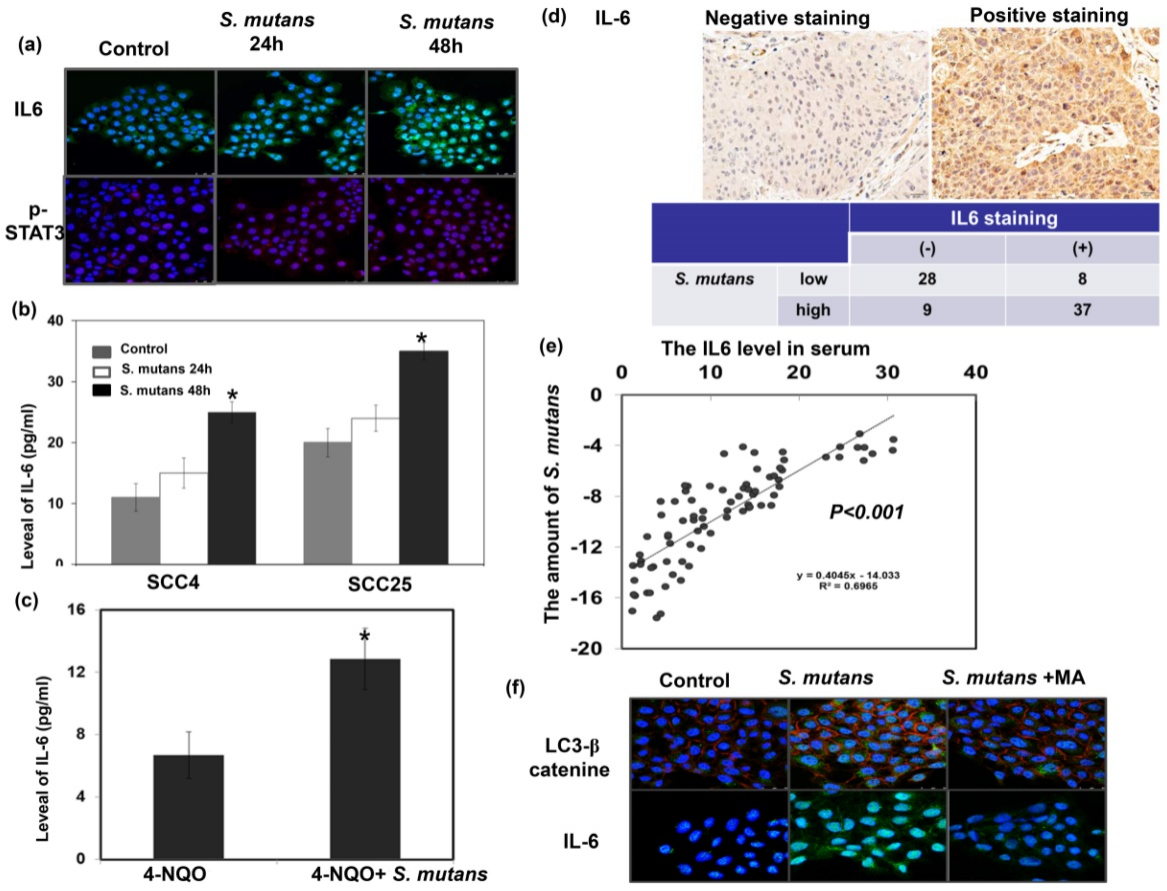
***S. mutans* infection is associated with enhanced IL-6 expression in oral cancer cells. (a)** IL-6 and p-STAT3 levels were evaluated by immunofluorescence. Representative slides are shown (DAPI, blue; IL-6, green; p- STAT3, red). **(b)** IL-6 levels in the supernatant of *S. mutans* -infected cells, as determined by enzyme-linked immunosorbent assay analysis. Data are presented as means ± standard deviations from three independent experiments; *, *p*<0.05. **(c)** *S. mutans* infection increased the level of IL-6 in murine serum noted in 4-NQO-induced oral cancer model. **(d)** The staining of IL-6 in human oral cancer specimens are positively associated with the presence of *S. mutans* infection. Representative images of positive and negative IL-6 staining are also shown. **(e)** Circulating IL-6 levels are positively associated with the presence of *S. mutans* infection. **(f)** Relationship among *S. mutans* infection, autophagy, and IL-6 signaling. Levels of LC3 I-II, and IL-6 in SCC4 cells were determined by immunofluorescence; representative images are shown (DAPI, blue; LC3/IL-6, green; β-catenin, red).

We previously reported that IL-6 overexpression was associated with poor prognosis in patients with HNC 16; moreover, the IL-6/ STAT3 signaling plays a critical role in CSCs and the EMT 17, 18. The IL-6/STAT3 pathway activation has been implicated in microbiome-induced tumor progression 5. Accordingly, we investigated the relevance of IL-6 signaling in the pro-OSCC effects of *S. mutans*. *S. mutans* infection induced IL6 expression in cancer cells; it also significantly elevated IL-6 levels in the cell culture supernatant and serum of 4-NQO-induced tumor model (Fig. 5a-c). Figure 5d-e demonstrated that the presence of *S. mutans* was associated with the increased IL-6 levels in cancer specimens and peripheral circulation. *S. mutans*-infected cells exhibited elevated autophagy levels associated with IL-6 production, and the autophagy inhibitor 3-methyladenine (10 mM for 1 h) attenuated the expression of IL-6 (Fig. 5f). The data indicated that there was a positive link between IL-6 expression and *S. mutans* infection in oral cancer cells, and induction of autophagy might contribute to the elevated IL-6 production observed in S. mutans-infected OSCC cells.

### Role of IL-6 in the tumor invasiveness induced by S. mutans for oral cancer

**Figure 6:**
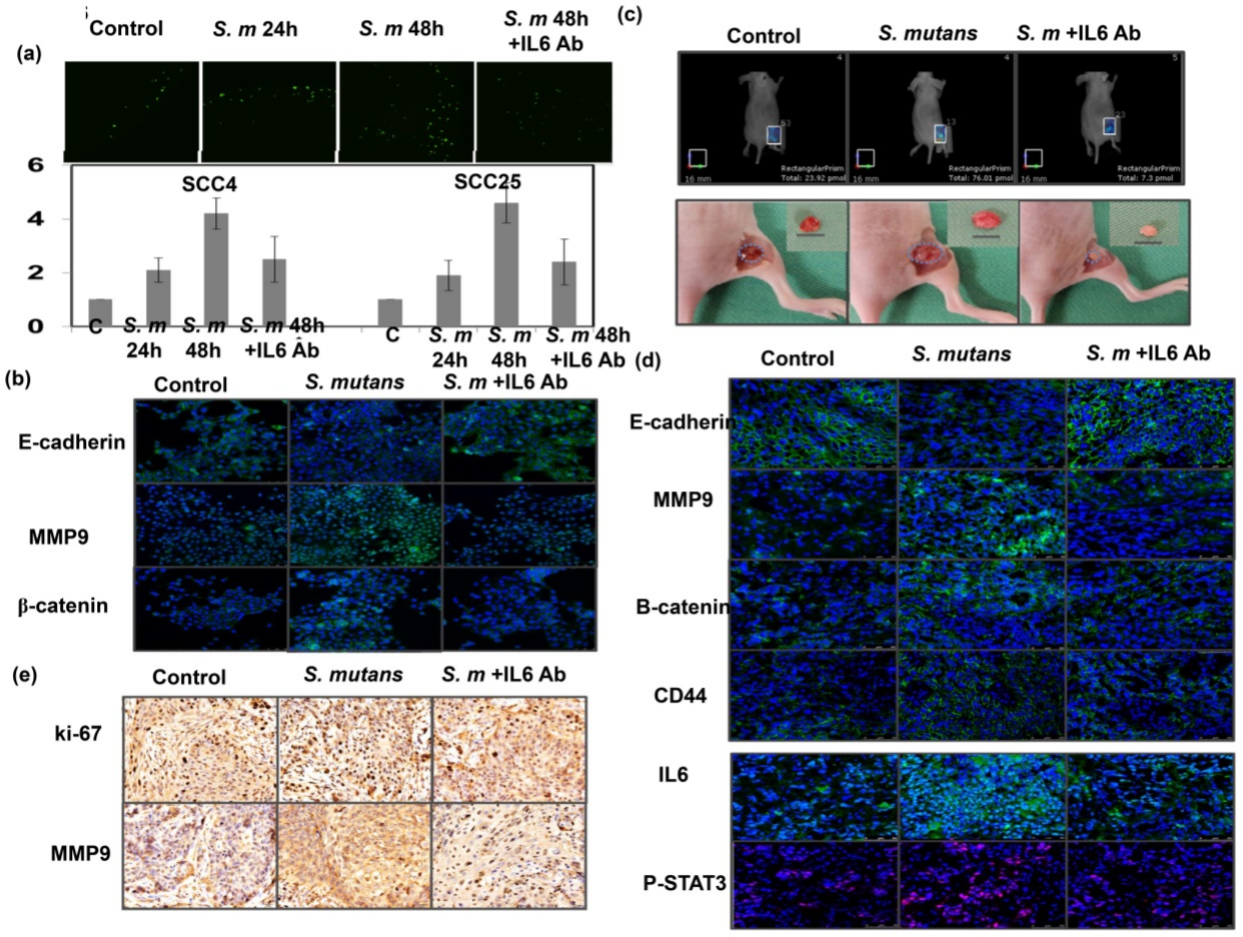
** Blockade of IL-6 signaling attenuates tumor invasiveness and stemness in *S. mutans* -infected oral cancer cells. (a)** Effects of IL6 inhibition on cell invasion in *S. mutans infection* -infected oral cancer cells; y-axis shows relative ratio normalized to number of invading control cancer cells. **(b)** Effects of IL6 inhibition on expression of EMT-associated proteins were determined by immunofluorescence. Moreover, effects of *S. mutans* infection on oral tumor growth in xenograft tumor model were determined by FMT combined with subcutaneous tumor growth** (c)**, the levels of EMT-related proton by immunofluorescence **(d)**, and evaluated by IHC **(e)**.

We further examined whether IL-6 inhibition could attenuate the effects of *S. mutans* on tumor aggressiveness. We found that IL-6 inhibition by *in vitro* treatment with anti-IL-6 for 48 h attenuated oral SCC cell invasion; it also suppressed the expression of EMT markers in S*. mutans*-infected OSCC cells (Fig. 6a-b). We established an orthotopic xenograft model to examine the role of *S. mutans* infection on human oral tumor growth and its relationship with IL6 signaling. As shown in Fig. 6c-e, *S. mutans* infection significantly increased glucose uptake; it also was linked to the augmented tumor growth and EMT changes. Furthermore, the data *in vivo* confirmed the findings *in vitro* and suggest that attenuating IL-6 signaling reverses EMT, CSC development, and tumor invasiveness in *S. mutans*-infected oral cancer cells.

## Discussion

The oral bacterial microbiome has been implicated in the development of dental caries and carcinogenesis 6, 7, 19. There have been studies reporting alterations in the microbiome of head and neck cancer (HNC). Poor oral health conditions, including chronic periodontitis and gingivitis, may result in a change in the oral microbiome leading to chronic inflammation and cancer progression 2, 20, 21. The opportunistic pathogens increase in the oral cavity when the oral equilibrium is disturbed, and have capability to spread disease 22. The changes in the relative abundance of some oral bacteria have been observed in HNC patients 23. It has been demonstrated that raised concentrations of saccharolytic and aciduric species, such as Streptococcus mitis/oralis, were significantly more prevalent on tumor surface than non-tumor tissue. Whether the abundance of these micro-organisms has any significance on carcinogenic process remains a concept worthy of further investigation. Microbial metabolites can contribute to inflammatory status and can influence the balance of proliferation and cell death in tissues. This can eventually progress to disorganized oral cell proliferation and cytoskeletal abnormalities 24. The induction of matrix metalloproteinase MMP9 can degrade the basement membrane and allow for an invasive and aggressive picture of OSCC. Changes in human microbiome could determine an oncogenic effect by the release of cellular anti-apoptotic signals, MMP9 expression, the induction of host epigenetic alterations and chronic inflammatory response, and modulation of anti-cancer immunity 23, 24. In this study, we sought to characterize oral microbiota dysbiosis in oral cavity SCC and its interactions with clinical outcomes. We found a significant differences in microbiome composition between OSCC and healthy samples. By microbiome analysis, we found that the presence of *S. mutans* in dental biofilm samples was more frequent in patients with OSCC than in healthy individuals. Furthermore, *S. mutans* 16S rRNA was more abundant in OSCC specimens than in non-malignant tissues. Research suggested the ongoing inflammatory response instigated by periodontal pathogens leads to an increase risk of chronic disease and cancers 25, 26. Our findings revealed the specific *S. mutans* significantly associated with OSCC risk. Of the bacteria believed to be pathogenic in periodontal disease and dental caries, *S. mutans* is a cariogenic organism that has a unique virulence property, and recently evolved to become a risk factor in the cancer etiology. *S. mutans* can survive under acidic environment and is considered to be one main organism for the decay of teeth 12, 22. Once established well in dental caries or around the dental prosthesis, *S. mutans* uses virulence factors associated with the carcinogenicity 27-29. The induced changes included affecting with cell-cell adhesion and enhance the process of tumor cell dissociation and invasion. Studies has indicated that high proportions of cancer patients carry *S. mutans* in their oral cavities and are at a greater risk of developing more complex oral implant infections which increase with cancer therapy 22, 27. Though *S. mutans* virulence and pathogenesis are characterized, it is still limited to the status of its carriage and virulence in patients with oral cancer. Thus, in the present study, role of *S. mutans* in OSCC was further evaluated. By analyzing the relationship between *S. mutans* and oral cancer in clinical patients. *S. mutans* infection was associated with advanced stage and poor disease control. Administration of the carcinogen 4-NQO in mice effectively induces oral cancers that closely resemble early human lesions 30. Accordingly, we further examined the effects of *S. mutans* on the induction of oral invasive tumor using the 4NQO-induced oral cancer model. In 4NQO-treated mice, *S. mutans* infection was associated with enhanced glucose uptake in oral lesions and elevated incidence of invasive carcinoma development. EMT and cancer stemness are essential for the progression of epithelial tumors 17, 31. Literature reports glycans produced by *S. mutans* affect with cell-cell adhesion of host through influencing the level of functional E-cadherin 27. In this study,* S. mutans* infection promoted EMT changes and induced the expression of CSC markers in oral cancer cells. These findings suggest that the presence of *S. mutans* plays a crucial role in oral cancer development and progression.

A number of mechanisms have been suggested through which human microbiota contributes to the cancer development such as inflammation generation, transfer of tumor-vulnerable phenotype, immunosuppression, induction of protumorigenic environment 4, 5, 8, 23. The oral microbiome not only drives the chronic inflammation that may precede OSCC, but is also involved in the direct influence on the host cell response. Local immunosuppression can be one cause of a more novel and pathogenic microbiome picture. The microbiota is able to regulate the tumor microenvironment via modulating the tumor-infiltrating myeloid cells. It has been reported that there was an association of M2 macrophage with oral tumorigenesis in the 4-NQO murine model. Interactions between the microbiota and the immune system are believed to impact on cancer immune surveillance. In dysbiotic states, microbiota affects inflammatory responses through inducing the production of inflammatory factors such as IL-6, and subsequently accelerates tumor progression. The activated IL-6 pathway has been implicated in microbiome-induced tumor progression 5, 32. In our previous study, elevated IL-6 levels were associated with poor prognosis in patients with HNC 16. We also found that IL-6 induced the expression of CSC- and EMT-related markers, while establishing an immunosuppressive tumor microenvironment. In the present study, we demonstrated a positive association between the presence of* S. mutans* and IL-6 levels in specimens from patients with OSCC. Furthermore, *S. mutans* infection was associated with higher IL-6 expression and enhanced recruitment of MDSCs in the 4NQO-induced tumor model.

In this study, we found that IL-6 production was induced in *S. mutans* -infected oral cancer cells. Furthermore, *S. mutans* infection was associated with MDSC recruitment, thus allowing cancer cells to escape from host immune responses. Conversely, blockade of IL-6 abrogated the induction of EMT- and CSC-related alterations in* S. mutans* -infected cancer cells. Based on these findings, we presume that *S. mutans* was involved in oral cancer development and progression in an IL-6-dependent manner. In the present study, the oral health state including the number of decayed teeth and periodontal survey records from the human cohort was lacking. Therefore, further work regarding the issue in a prospective study is needed to elucidate the role of *S.mutans* as a pathogen related to oral health and being an important contributor to oral carcinogenesis.

## Conclusion

The presence of *S. mutans* promoted oral cancer development and progression, at least partly by increasing IL-6 production. Therefore, good oral hygiene and targeting IL-6 signaling should be implemented in current treatment approaches for *S. mutans* -positive patients with OSCC.

## Funding

The work was support by Chang Gung Memorial Hospital. Grant CMRPG6L0161-2 (to Y.Y. Chen).

## Figures and Tables

**Table 1:** The Clinical characteristics of patients with OSCC

	No. of patients		
	Low S. mutans	High S. mutans	* p* value
**Patients**	36	46	
**Age**			
Range	25.8~76.13	37.7~82.4	0.332
Median	56.4	57.6	
**Differentiation**			
WD~MD	26	22	0.026*
PD	10	24	
**Clinical stage**			<0.001*
I-II	19	6	
III-IV	17	40	
**IL-6 staining**			<0.001*
negative	28	9	
positive	8	37	
**Serum IL6**			<0.001*
mean	5.25	16.39	
SD	0.48	0.98	
**Disease failure**			0.001*
No	27	19	
Yes	9	28	
**Overall survival**			0.036*
Alive	32	32	
Dead	4	14	
